# Acting on audit & feedback: a qualitative instrumental case study in mental health services in Norway

**DOI:** 10.1186/s12913-018-2862-y

**Published:** 2018-01-31

**Authors:** Monica Stolt Pedersen, Anne Landheim, Merete Møller, Lars Lien

**Affiliations:** 1 Norwegian National Advisory Unit on Concurrent Substance Abuse and Mental Health Disorders, Innlandet Hospital Trust, P.B. 104, 2340 Brumunddal, Norway; 2 Faculty of Medicine, University of Oslo, Oslo, Norway; 3 Norwegian Centre for Addiction Research, University of Oslo, Oslo, Norway; 4 Østfold Hospital Trust, Grålum, Norway; 5 Inland Norway University of Applied Sciences, Campus Elverum, Elverum, Norway

**Keywords:** Audit and feedback, Implementation, Mental health services, Co-occurring disorders, Qualitative methods, Case studies

## Abstract

**Background:**

The National Guideline for Assessment, Treatment and Social Rehabilitation of Persons with Concurrent Substance Use and Mental Health Disorders, launched in 2012, is to be implemented in mental health services in Norway. Audit and feedback (A&F) is commonly used as the starting point of an implementation process. It aims to measure the research-practice gap, but its effect varies greatly. Less is known of how audit and feedback is used in natural settings. The aim of this study was to describe and investigate what is discussed and thematised when Quality Improvement (QI) teams in a District Psychiatric Centre (DPC) work to complete an action form as part of an A&F cycle in 2014.

**Methods:**

This was an instrumental multiple case study involving four units in a DPC in Norway. We used open non-participant observation of QI team meetings in their natural setting, a total of seven teams and eleven meetings.

**Results:**

The discussions provided health professionals with insight into their own and their colleagues’ practices. They revealed insufficient knowledge of substance-related disorders and experienced unclear role expectations. We found differences in how professional groups sought answers to questions of clinical practice and that they were concerned about whether new tasks fitted in with their routine ways of working.

**Conclusion:**

Acting on A&F provided an opportunity to discuss practice in general, enhancing awareness of good practice. There was a general need for arenas to relate to practice and QI team meetings after A&F may well be a suitable arena for this. Self-assessment audits seem valuable, particular in areas where no benchmarked data exists, and there is a demand for implementation of new guidelines that might change routines and develop new roles. QI teams could benefit from having a unit leader present at meetings. Nurses and social educators and others turn to psychiatrists or psychologists for answers to clinical and organisational questions beyond guidelines, and show less confidence or routine in seeking research-based information. There is a general need to emphasise training in evidence-based practice and information seeking behaviour for all professional groups.

**Electronic supplementary material:**

The online version of this article (10.1186/s12913-018-2862-y) contains supplementary material, which is available to authorized users.

## Background

Audit and feedback (A&F) is often the starting point of quality improvement projects. It aims to close the gap between recommended and actual practice. A&F may be defined as a ‘summary of the clinical performance of healthcare provider(s) over a specified period of time’ [1]. It can be a useful intervention to improve health professionals’ compliance with desired practice and is one of the most widely used strategies for improving practice [1–3]. A&F may be described as a circular process with several stages [4, 5]. It is often designed to be a part of a multifaceted improvement strategy, where audit with feedback is theorised to promote health professionals’ motivation to improve practice [1, 6–8]. Reflecting on results, agreeing on where improvement is needed and producing an improvement plan are essential components of the process.

The past 10–15 years have seen an increasing interest in guideline implementation strategies [9]. The Norwegian National Health Plan (white paper) states that evidence-based practice is a goal in Norwegian health policy [10]. Health authorities produce clinical guidelines in order to encourage a more evidence-based practice and more harmonised services [6]. Clinical guidelines give recommendations for best practice and may be used as benchmarks against which clinical practice may be evaluated [11]. The Norwegian Directorate of Health is the only organisation with a mandate to develop and disseminate national clinical guidelines in Norway. Recommendations in national guidelines are not legally binding (unless tied to a legal act), but normative by pointing to the desired and recommended courses of action [12].

Several studies show high co-occurrence between substance use disorders and mental health problems. This is well documented through clinical and epidemiological studies [13–17]. “The National Guideline for Assessment, Treatment and Social Rehabilitation of Persons with Concurrent Substance Use and Mental Health Disorders” [18] (hereafter the National Guideline) was launched in March 2012 and as one of several initiatives designed to improve services for people with concurrent substance use disorders and mental illness (see Additional file 1). The Norwegian National Advisory Unit on Concurrent Substance Abuse and Mental Health Disorders (hereafter the National Advisory Unit) has developed a standardised electronic audit questionnaire mirroring the recommendations in the National Guideline [19]. This is a pre-determined audit aimed at District Psychiatric Centres (DPC), to support implementation of the National Guideline, and to be used together with an action form.

Despite broad agreement on the importance of guidelines, they are not always easily translated into practice [20], often referred to as barriers to change [21]. Several strategies and theories exist as to how recommendations from research might be implemented [22–24], including process models aimed at describing and guiding the process [25]. A recent Cochrane Review [26] was unable to identify the effectiveness of implementation strategies in mental health care. The Effective Practice and Organisation of Care (EPOC) Group is a Cochrane Review Group [27], whose tasks include reviewing implementation strategies aimed at health professionals. One of the implementation strategies are A&F [1]. A key function of A&F is to identify sub-optimal performance and recognise the need for change [8, 28, 29]. Studies show that the effect of A&F on professional behaviour and patient outcome ranges from little or no effect to a substantial effect [1, 30] and this may be due to the characteristics of the behaviour it is targeted at, the healthcare staff audited, their context, the patients/consumers, or the components of the intervention itself [31, 32]. A&F may be most effective when the research-practice gap is large, the person responsible for the A&F is a supervisor or colleague, it is conducted more than once, it is given both verbally and in writing, and it includes clear targets as well as an action plan [1, 29]. We still do not fully understand the key ingredients of a successful A&F intervention or the mechanisms of action of effective A&F interventions in healthcare [7, 29]. Most of the research concerns the effect of A&F and how and when feedback is given. To our knowledge, less is known about how health care professionals discuss and use the results from the A&F when they meet in quality improvement (QI) teams with the purpose of selecting improvement areas, i.e. acting on the results from the audit with the purpose of improving service quality in a mental health care setting.

The aim of this study was to describe and investigate what is discussed and thematised when QI teams in a DPC work to complete an action form as part of an A&F cycle.

## Methods

**Fig. 1:**
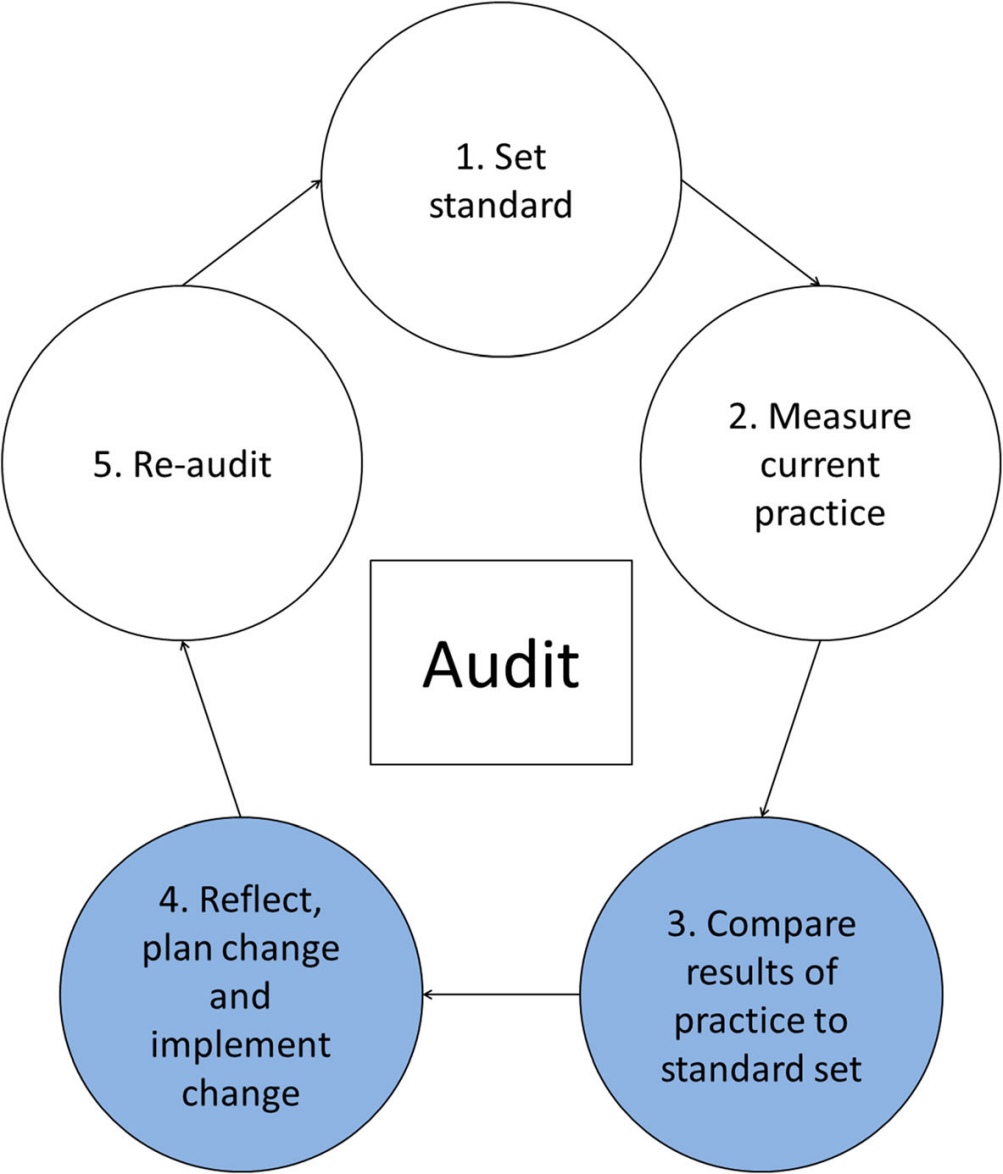
An audit-cycle

The process described followed a common A&F cycle, and this study involved Phases 3 and 4; the audit had been completed, feedback had been given, and QI teams met to fill out action forms (see Fig. 1).

### Design

This was a qualitative, instrumental, multiple case study [33, 34]. The phenomenon we sought insight into was part of an improvement process: how QI teams discussed results from A&F and completed action forms in a “natural setting”.

### Study site

The study took place in a DPC in South-Eastern Norway. The DPC is part of a larger hospital trust and consists of four units, two outpatient units and two inpatient units, each representing a case; where specialist mental health services are offered to approximately 72,000 inhabitants. Each unit is subject to various organisational and professional frameworks and is thus considered a separate case. Meanwhile, they belong to the same DPC, with the same management and the same organisational and legal conditions providing a common external conceptual framework connecting the cases and the activities involved.

The two inpatient units had similar staff in terms of number and type and length of education. In Norwegian inpatient units, nurses and social educators (minimum three-year bachelor’s degree) and assistant nurses are commonly referred to as milieu personnel, while psychologists and psychiatrists are often referred to as therapists. The units had somewhat different groups of patients; one had more patients with psychosis-related disorders and another had more patients within general psychiatry or personality disorders. The staff consisted mainly of milieu personnel and 1–2 therapists in both inpatient units. One outpatient unit was a crisis resolution team (CRT), mainly consisting of staff like nurses, social educators or others with similar relevant qualifications, such as social workers, and had a psychologist and psychiatrist attached to the unit. They reached out from the hospital setting to patients mostly referred from GPs or other hospital departments. The second outpatient unit was mainly staffed by therapists such as a psychiatrists, psychologists or specialist nurses, operating as a general psychiatric outpatient clinic with regular hours and booked appointments.

### Recruitment and implementation process

An improvement process for the entire DPC was proposed by the leader group of the Hospital Trust in October 2013 and thereafter agreed upon by the DPC later the same year, with an anticipated start in the units in the beginning of 2014. The decision was to implement the National Guideline guided by the implementation process outlined by the National Advisory Unit. The DPC was selected since they were ready to start an improvement process and we were looking for a site to study the implementation process. The implementation process was owned and executed by the DPC and would have taken place without the research project.

The National Advisory Unit had developed tools to support implementation of the National Guideline as part of the development of the guideline. One was a brief description of an implementation process in stages adapted from the implementation of change model of Grol and colleagues [23]. This was accompanied by an electronic survey to audit practice in DPCs and a standardised action form with a template of how to use it. The action form contained columns for areas of improvement, goals, actions, progress plan, main responsibility, economic assessments and evaluation. The survey contained 46 questions about screening practices, assessment of target group, integrated treatment, collaboration, use of evidence-based methods and competence requirements, and was a self-report questionnaire. The audit survey was designed to be applicable to mental health services and was available with templates at the National Advisory Unit web site [19].

An audit of existing practice was conducted in February and March 2014. A project supervisor (MM) from the Health Trust, with special responsibility for concurrent substance abuse and mental health disorders, assisted in the execution of the audit. All four units of the DPC were included in the audit. Feedback of the results was given verbally and in writing by the project supervisor to each unit separately at meetings for the whole unit with unit leaders present. Results were presented at unit level, together with sessions on evidence-based practice, recommendations in the National Guideline and how to conduct an improvement process informed by the Knowledge-to-Action cycle [35]. The meetings were led by the project supervisor in March 2014.

The DPC formed QI teams from each unit to facilitate the process. Each unit leader selected who should participate in the group. Seven QI teams were set up with participants and meeting schedule, to start about one month after the audit results were presented. The participants in the teams reflected the staff in each unit in terms of education and job position as described above. Only one of the teams (from the CRT) had the unit leader present at QI team meetings, the teams from the general outpatient units had an appointed leader with senior experience, and the inpatient unit teams had a more flat leader structure. A total of 11 meetings were held (see Table 1).

**Table 1:** Quality Improvement teams in the DPC. Numbers of teams, team members and meetings

	QI teams	Participants in QI teams	Meetings
General outpatient clinic	2	14	3
Inpatient unit 1	3	21	3
Inpatient unit 2	1	6	1
Crisis Resolution Team (CRT)	1	12	4
Total	7	53	11

Meetings of the QI teams were held in April and May 2014. The purposes of the meetings were outlined by the National Advisory Unit [36]:

1. to discuss the results of the audit and identify any gaps between the recommendations of the guideline and local practice
2. to choose areas for improvement on this basis
3. to discuss local context and barriers and enablers for the improvement areas
4. to choose suitable actions for the goal of improvement
5. to discuss how to monitor and evaluate

The discussions in the QI meetings were based on the collected audit data and the participants’ professional and organisational experience. A joint action plan for the DPC was adopted in June 2014 with common areas for improvement, actions, work schedule, responsibilities, budget assessment and evaluation, based on the work conducted in the QI teams. Three improvement areas were selected: 1) Screening the use of substances with AUDIT (Alcohol Use Identification Test) and DUDIT (Drug Use Identification Test), 2) Enhancing knowledge of the treatment of concurrent substance abuse and mental illness, and 3) Strengthening integration between the DPC, substance abuse departments and community health care. The actual implementation phase was planned for autumn 2014 to spring 2015, with a re-audit in spring 2015.

One researcher (MSP) spent some time at the study site before the actual data collection started, attending management meetings and talking to the project supervisor, the unit leaders and the department manager. This was to gain an understanding of the general context of the cases.

### Data collection

All seven QI teams, with eleven team meetings, were observed, resulting in 11 h of recordings. The meetings lasted between 36 min and 1 h and 52 min. All were held at the hospital, in familiar environments for the teams.

In this study, we mainly used open non-participant observation to describe and investigate an early stage of an improvement process: how and what the staff discussed when aiming to adopt the recommendations in the National Guideline by filling out the action forms as part of completing the audit cycle. To be able to record words rather than summaries, we used an audio recorder together with the observer’s reflection notes. These were always written on the same day as the meeting, to ensure fresh reflections on the atmosphere, surprising statements and agreements or disagreements in the teams.

One observer (MSP) was present at the meetings. The researcher was unfamiliar with the DPC and its staff before the process started. It was considered important to explain that the researcher was not the one to initiate or decide on the improvement process or to perform the A&F. At her first meeting with a team, the researcher introduced herself, shook hands with all the participants and presented the research project in detail. The researcher has a background as an information scientist in medicine and organisational learning, with a focus on evidence-based practice and implementation. The observer sat at the table together with the participants in the meeting. This enabled open observation without unduly disturbing the process.

### Data analysis

The audio recordings from the meetings were transcribed verbatim and in detail. Together with the reflection notes, this formed the data base. Such an analysis relies largely on the spoken word, hence the use of an audio recorder. But the reflection notes were also part of the analysis, to make sense of the atmosphere in the room, the relationship between the participants, etc., and to observe what was not verbalised. The computer software QSR International’s NVivo 10 was used to help to organise the analytical process.

A short, but thorough description of the cases was written, together with necessary context.

The analysis was performed in six steps as a thematic analysis suitable for instrumental case studies [33, 37]. Each transcription and reflection notes was read and listened to several times. Codes were assigned to meaningful units in the text, rather generously to ensure that nothing interesting or important was lost at this stage. The codes identified semantic features of the data. Codes were assigned to the data base, case by case. A thorough review of the codes was undertaken, with uncoding and recoding while comparing with the text, to ensure a unified way of labelling and interpreting the text. The third phase was to start searching for themes, by sorting codes into potential themes. The codes could appear across the cases, but were sometimes apparent in just one case and gave a first glance at similarities and differences in the cases. We sought to ascertain whether the codes could form a theme, going back and forth between potential themes, the codes and the dataset. Phase four consisted of reviewing and refining the themes. At this phase, all the data was reread to ensure that the themes fitted with the dataset. In phase five, we defined and named the themes. Phase six was to produce the report. We made adjustments until the end to make sure the analysis and themes had captured the essence.

In a multiple instrumental case study, we are interested in understanding the phenomenon under study [38]. We sought to shed light on the research question by using multiple cases bound together by organisational belonging and the common activity undertaken. We needed to strike a balance between the particular and the common features of each of our four cases while looking for similarities and differences. We mainly reported on general perspectives where present, in order to form an idea of the use of an audit in a DPC as a whole [33, 38, 39].

## Results

The aim of this study was to describe and investigate what is discussed and thematised when QI teams in a DPC work to complete an action form as part of an A&F cycle. The results of the audit showed a mismatch between recommendations in the National Guideline and local practices in several areas. This applied to all units, albeit somewhat differently within the various topics in the audit.

A whole range of issues regarding daily tasks, leader engagement, organisational issues at the unit or the DPC, cooperation, responsibilities and busy schedules were up for debate when the QI teams gathered to discuss their audit results. The audit seemed to prompt much more than just finding improvement areas and filling out action forms. Acting on the audit may thus be seen as important for various purposes, such as allowing for in-depth discussions on different aspects of work.

### Eye-opening and sharing practices

When the QI teams gathered, some began immediately to complete the action forms and came back to the audit results afterwards, but most devoted time to the actual process of reflection and discussion of their own unit’s results. They focused on the results showing the largest gaps between recommended and actual practice. It was a realisation of a research-practice gap, an eye-opener.

The staff seemed to become aware of their own practice in a different way through the discussions. Important events in some of the meetings were participants asking about and listening to details of colleagues’ practices. “How do you do this?”, “Are you using the recommended questionnaire or do you ask questions more randomly?” are examples of comments when they began to delve into their own practice. Sometimes they seemed somewhat surprised when listening to each other’s stories about how they perceived everyday tasks or actually performed them in detail. It became clear that practice could sometimes develop in different directions, and that the staff did not necessarily have the same knowledge base for their practice.

Many participants realised for the first time that there existed a standard, i.e. a national guideline; a typical statement was “I wasn’t aware there was a standard for this, I’ve only done what seemed best or what was closest to my previous knowledge or educational background”. Several times there were statements about the newly discovered gap, but they would also literally point at the audit results, holding them up like a proof of practice – or lack of best practice. This awareness seemed to be important in enabling the start of an implementation process, or choosing improvement areas and filling out the action forms as required, representing willingness to take action.

### Lack of knowledge and unclear role expectations

Without exception, all QI teams acknowledged their lack of knowledge about the patients with concurrent substance use disorder and mental illness, although some members of staff had experience and expertise in the field. Several of the teams talked about lack of knowledge about substances, their impact and what signs to look for in patients in active intoxication, withdrawal phases or long-term effects.

QI teams from three of the units seemed to be uncertain about the expectations placed on them. What tasks were they expected to perform and what expertise was required in implementing recommendations from the National Guideline? A decision on screening for substance use as an improvement area arose fairly early in the discussions, followed by the question “What do we do when we know?”, i.e. “How do we deal with the awareness that a patient has a concurrent substance use disorder and mental illness?”

In all teams there were discussions related to uncertainty about the participants’ own competence, but they manifested themselves differently in the general outpatient clinic on the one hand, and in the three remaining units on the other. In the latter, the QI teams appeared mainly to be uncertain about what they were supposed to know and work expectations, and we found that the temperature in the discussions sometimes rose concerning expectations from the management and we could see signs of despondency and even anger. These statements usually came after recognition of the research-practice gap from the audit. Some of the employees seemed to feel taken by surprise by the gap, or felt that they should have known already and taken care of this and that this responsibility lay with the management.

We also found insecurity around whether they were actually giving the best treatment to the patients with co-occurring disorders. So when they discussed the audit results, their own practice, and implementation of new recommendations, we noted statements and reflections that what they were used to doing was no longer valid or good enough. We often found that the staff demanded in-service courses and seminars, and stated that they should have been offered this earlier. It was clear that the statements about lack of knowledge arising from the audit results led to a decision to gain enhanced knowledge of the treatment of concurrent substance abuse and mental illness.

The QI teams from the general outpatient clinic also recognised a lack of knowledge in the field of substance use disorders. Otherwise, they recognised themselves as professionals. Their uncertainty was related to whether their expertise was acknowledged by the management and taken into account as they felt it should be. They talked about “we, as professionals”, and we could trace dejection and slight defiance when this topic was discussed in both teams from the general outpatient clinic.

### Where to seek answers to clinical questions

The psychiatrists and psychologists seemed to be the ones to “own” the knowledge. They were the ones to keep themselves updated by virtue of education or position. Then the nurses, assistant nurses and social educators could harvest from this knowledge. We found this phenomenon to be present when they were looking for someone to hold educational courses and when they addressed clinical questions to colleagues, and also when they referred to where or whom they got knowledge from. When discussing “best practice”, statements like “psychiatrists have different opinions” appeared. Milieu personnel, nurses and social educators seemed to be used to seeking answers from psychiatrists or psychologists, but found it frustrating when they had divergent opinions on the same matter. It seemed to give rise to confusion and professional insecurity.

None of the professions or QI teams talked about evidence-based practice, systematic reviews or the fact that knowledge might be found in the hospital’s medical library. Some of the teams talked about “building a library”, but less about what should be in it other than certain academic textbooks. There were, however, in some teams references to the National Guideline as a source of knowledge of the area, and it was also held up like a “proof of best practice” with statements like “This is the knowledge base” in a couple of teams. But there were also those who clearly questioned the National Guideline as trustworthy or as a source of best practice.

In the QI teams formed by the outpatient clinic, the discussions were somewhat different. They acknowledged a professional disagreement, and solved the disagreement by either accepting a professional discussion or by simply sweeping the whole disagreement under the rug and accepting differences in practice, mainly due to differences in specialities.

### New practices in old systems

All QI teams talked about how the recommendations fitted in with their usual practice and that new ways of working and new practices often felt fragmented and not integrated with current practice. They used the A&F to discuss local practice and possible barriers to new practice, like resources, knowledge of substance abuse, organisation of daily tasks, and lack of time. They assumed that if they put the National Guideline recommendations into practice, it would be uncertain how this would fit in with their usual practice, the way the unit and its tasks were organised – and the way the DPC works. This was perceived as a clear barrier to implementing the recommendations from the Guideline.

At a system level, there were statements about the lack of connected and integrated treatment. Seven questions in the audit survey concerned cooperation and integrated treatment. These particular questions generated discussion in most of the QI teams. All the teams realised that they did not cooperate to any great extent with others, whether GPs, local authorities or substance abuse treatment units. They found that patients with the most severe co-occurring disorders fell between cracks also at a system level, and were thus not receiving appropriate treatment. The audit results were used to discuss how the system worked, especially for people with concurrent substance use disorder and mental illness. Several teams, particularly from the inpatient units, stated that they did not know the system they were working within well enough, and were therefore unable to offer integrated or optimal services, leading to interrupted and fragmented chains of treatment. This was clearly seen as a barrier, but not one they could easily find a solution to. It was considered a management or organisational problem.

## Discussion

We found that acting on A&F stirred up a wealth of discussions around local practice, organisational issues and responsibilities and was also used as a way of showing resistance to management. The QI team meetings were important to gain awareness of local practice and to recognise a lack of knowledge on substance abuse and a neglected group of patients, namely those with concurrent substance use disorder and mental illness, and also to identify unclear role expectations. Health professionals such as nurses, social educators or nursing assistants usually seek knowledge from psychiatrists or psychologists, but get frustrated when the answers diverge depending on whom they ask. There were concerns about whether new tasks arising from the guideline recommendations would fit in with the organisation’s usual way of working and current resources.

Our research concurs with earlier research on quality improvement from various areas of health services [40–42] in that working with an A&F cycle involving completion of action forms in QI meetings was a useful, welcome and necessary opportunity to discuss practice amongst practitioners also in mental health services. It seemed to open the door to negotiations on improvement areas, task allocations and multidisciplinary work and could be considered important steps on the way to implement recommendations from the National Guideline.

Acting on A&F created an awareness of local practice and a standard for practice in a national guideline which is in line with the intentions of an A&F process: to prompt the need for change [2, 29]. Although the team members were not instructed to look for the largest gaps in the audit feedback, the QI teams usually ignored the results showing that practice was more or less in line with the guideline, and acted upon the greatest discrepancies, as also shown in previous studies [1, 8, 28, 29]. As an example, the QI team acted upon results showing gaps in the use of screening tools for detection of substances, and in general knowledge about substances and substance abuse treatment. There were no benchmarked data from other services or national audits to compare the audit results with, and the QI teams therefore discussed the data in a subjective manner. Self-assessments are subjective to bias [43], and may therefore give poor data quality. We might question whether the addition of audit data from patient records could have given better and perhaps different results, providing a richer and more accurate picture and adding to the team discussions more usefully than self-completed questionnaires alone. However, audits from patient record alone would possibly not give sufficiently accurate data, due to inadequate patient record keeping, reported by the Norwegian Board of Health Supervision [44] and the Norwegian System of Patient Injury Compensation [45]. Studies show that self-assessments contribute to professional development, learning and practice change [46] and are thus a valuable tool. All of the teams had senior staff present and representatives from various professions, which gave the team broad competence and experience of practice and context and added credibility to the meetings and discussions.

Our study found a lack of knowledge on substance related disorders and unclear role expectations among the health professionals. Other studies have found that for example nurses experience a lack of alcohol-related knowledge and skills, which was considered a barrier to the implementation of screening tools [47], while participants in a Swedish study asked how they were supposed to change clinical practice when they did not have adequate skills to use recommended tools [48]. The lack of familiarity with the guideline and lack of faith in one’s own influence on the quality of care reported in the feedback are known barriers to implementation [32, 49]. The National Guideline came with a strong emphasis on concurrent substance use disorder and mental illness and placed greater responsibility on mental health care for patients with substance use disorders. Tasks and responsibilities, for example screening with appropriate tools to detect substance abuse, previously seemed to lie with one professional group in the DPC, but were now expanded to a larger group of professionals, from only psychologists or psychiatrists to also include nurses or social educators. From other studies we know that interchangeable roles and responsibilities may cause tension and power struggles, but more often interprofessional collaboration is beneficial for team functioning [50]. For collaboration to be successful, all team members should have a sense of autonomy, suggesting independent and self-determined practice to ensure a true complementary contribution. We may question whether job expectations and professional roles were inadequately formulated from leaders to all professional groups, particularly in the inpatient units. It might also be a general lack of arenas to discuss practice and to develop collaborative practices. Discussions concerning roles and responsibilities might have been more successful if unit leaders had been present at all meetings. Support from management or inspirational team leadership appear to be important in QI teams, bringing different professionals together [51]. We might also speculate on whether the staff are disclaiming responsibility in not taking national guidelines into account or not readily taking on the responsibility of new tasks or roles in the QI meetings. These issues might not easily be solved with educational courses or improving clinical skills alone, but more general with empowerment of all health professionals in autonomous contribution to collaborative work, which is also addressed by other authors [50, 52].

The psychiatrist/psychologist group had legal authority for decision making and patient treatment responsibility, but our findings seem to show that the responsibility was expanded to a “definition of knowledge”. This could be a natural consequence of the responsibility associated with the psychiatrist in a unit (as medical doctors are the only profession legally entitled to prescribe medication). Nurses, social educators, etc. approached the group of psychiatrists and psychologists instead of their own professions or the “evidence”, i.e. clinical guidelines or research, for support in their practice. We know from international studies that physicians and nurses pursue roughly half of the questions they raise in clinical practice [53], and colleagues are a preferred source for answers [53–55]. Information-seeking behaviour may vary between nurses and physicians; nurses more often ask a colleague for answers to clinical questions, while physicians more often turn to online databases [56]. A hierarchical system might help to explain the expansion to “all kinds” of knowledge, but also a lack of training in information-seeking behavior, maybe particularly in the group of nurses and social educators.

Integrated treatment requires staff to coordinate collaboration between service providers [57]. The feeling of a lack of connected and integrated services at a system level found at this DPC is not unfamiliar [58–62]. The National Guideline recommended integrated treatment, which was a responsibility of the DPC, and took a pragmatic approach in stating that different and independent entities should coordinate their services for patients in an orderly manner. The important factor is that the patient experiences the treatment as integrated and connected [18]. We know that aspects of the guideline itself can be a major barrier to its implementability [32]. The National Guideline was assessed for implementability by the GLIA instrument [63], and found to be not easily implemented due to somewhat general recommendations which were not necessarily easy to transfer to practice [18]. This is therefore a possible explanation for the feeling of lack of connected and integrated services.

### Strengths and limitations

A major strength of this study was the natural setting where the A&F cycle took place. This allowed for an exploration of what happened in the QI teams after A&F when the process was organised by the DPC itself, without a research team being part of the project, and thus reflected real people in real settings. In many similar projects, the research team has had an important and visible presence in all parts of the A&F cycle, from setting the standard, auditing practice, giving feedback and facilitating the work of the QI teams and in that way possibly having an impact on the process itself. Case studies are useful for gaining an in-depth understanding of an issue and answering the “how” questions when the behaviour of those involved cannot be, or is not desired to be, manipulated [33]. They focus on contemporary events in real-life contexts, where the phenomenon of interest is interdependent with the context of study [39]. An instrumental case is used to illuminate the “problem”; the case itself is not the most important part [33]. We use the case to understand and answer the research question and it is thus instrumental to accomplishing this understanding [34].

Qualitative research and case studies may be limited in their generalisability. A common conclusion in implementation research is that the effect of the context makes the findings less relevant in other settings. However, we believe that our study has relevance to other settings in and outside mental health care services, and knowledge gained from this study may contribute to the planning and execution of A&F cycles under similar circumstances.

There are certain considerations involved in choosing a multiple case study design. One is vulnerability, in that that the supposedly common cases may turn out to be less common than originally thought in certain aspects, and thus do not shed light on the research question as expected. However, we believe that the cases in this study are quite representative for many Norwegian DPCs in an initial phase of the implementation process, enabling generalisation to other DPCs. We have described the case in as much detail as necessary to account for this risk and make it transparent. A strength of the study is the use of instrumental multiple cases, allowing for a holistic view of the phenomenon, less dependent on the individual context. It is nevertheless worth noting that the cases are linked to a general context, the DPCs being similar in all cases.

There are strengths of open non-participant observation considered important enough to choose it as a method. Open observation allows for conceptualisation of health professionals behaviour and interaction in their natural environment [64] and the direct evidence of observed outcomes and processes, as opposed to reported accounts. It may also capture the flavour of the setting and provide specific examples. Open observation, not driven by theory, was enabled by the relatively demarcated units of analysis, the QI team meetings.

There is no doubt that both the use of an audio recorder and the presence of an observer are very noticeable elements in a meeting room (“the researcher effect”). It is uncertain whether the observer or the audio recorder is the more significant element for the participants. The observer not being a health professional, but with extensive experience from administrative parts of health services, gives a possibility for a fresh look at a health care setting, allowing for new insight. We believe the use of an audio recorder and reflection notes immediately after meetings helped to ensure the verifiability and thus the reliability of the observations.

## Conclusions

The aim of this study was to describe and investigate what is discussed and thematised when QI teams in a DPC work to complete an action form as part of an A&F cycle. The A&F was based upon current knowledge of the most effective interventions and pre-made tools to support the process. The study showed that acting on A&F provided a welcome opportunity to discuss practice in general, enhancing awareness of good practice. There was a general need for arenas to meet and discuss current practice, best practice and recommendations from guidelines and also how to meet divergent demands in an open and collaborative way. QI team meetings after A&F may well be a suitable arena for this. The study also showed that self-assessment audits seemed valuable, particularly in areas where no benchmarked data exists, and there was a demand for implementation of new guidelines that might change routines and develop new roles. QI teams could benefit from having a supportive leader present at team meetings to provide direction particularly on organisational questions, and team members might also benefit from a general empowerment to autonomous contribution to interprofessional collaboration.

Nurses and social educators turn to psychiatrists or psychologists for answers to clinical and organisational questions beyond the National Guideline, and show less confidence or routine in seeking research-based information. There is a general need to emphasise training in evidence-based practice and information-seeking behaviour for all professional groups. New guidelines will keep coming, putting new demands on staff in mental health care, and increased knowledge in these areas could hopefully lead to less insecurity about roles and practice, and to discussions of audit results based on research-knowledge familiar to all professional groups.

## Additional file

Additional file 1: Information on The National Guideline for Assessment, Treatment and Social Rehabilitation of Persons with Concurrent Substance Use and Mental Health Disorders. (DOCX 16 kb)

## Abbreviations

A&F: Audit and Feedback
AUDIT: Alcohol Use Identification Test
CRT: Crisis Resolution Team
DPC: District Psychiatric Centre
DUDIT: Drug Use Identification Test
QI team: Quality Improvement Team
The National Advisory Unit: The Norwegian National Advisory Unit on Concurrent Substance Abuse and Mental Health Disorders
The National Guideline: National Guideline for Assessment, Treatment and Social Rehabilitation of Persons with Concurrent Substance Use and Mental Disorders

## References

[1] Ivers NM, Jamtvedt G, Flottorp S, Young JM, Odgaard-Jensen J, French SD (2012). Audit and feedback: effects on professional practice and healthcare outcomes. Cochrane Database Syst Rev.

[2] Jamtvedt G, Young JM, Kristoffersen DT, O'Brien MA, Oxman AD (2006). Does telling people what they have been doing change what they do? A systematic review of the effects of audit and feedback. Qual Saf Health Care.

[3] Hysong SJ (2009). Meta-analysis audit and feedback features impact effectiveness on care quality. Med Care.

[4] Potter J, Fuller C, Ferris M: Local clinical audit: handbook for physicians. In: Healthcare quality improvement partnership. London: HQIP. 2010. https://www.hqip.org.uk/resources/hqip-local-clinical-audit-handbook-for-physicians/. Accessed 25 Jan 2018.

[5] Dixon N, Pearce M, Quest HQ: Guide to using quality improvement tools to drive clinical audits. In: Healthcare quality improvement partnership. London: HQIP. 2011. http://www.hqip.org.uk/resources/hqip-guide-to-using-quality-improvement-tools-to-drive-clinical-audit/. Accessed 2 Oct 2015.

[6] Grimshaw JM, Thomas RE, MacLennan G, Fraser C, Ramsay CR, Vale L (2004). Effectiveness and efficiency of guideline dissemination and implementation strategies. Health Technol Assess.

[7] Foy R, Eccles MP, Jamtvedt G, Young J, Grimshaw JM, Baker R (2005). What do we know about how to do audit and feedback? Pitfalls in applying evidence from a systematic review. BMC Health Serv Res.

[8] Burgess R (2011). New principles of best practice in clinical audit.

[9] Grimshaw JM, Eccles MP, Lavis JN, Hill SJ, Squires JE (2012). Knowledge translation of research findings. Implement Sci.

[10] Meld. St. 16 (2011-2015) (2011). Nasjonal helse- og omsorgsplan: 2011–2015 [National Health and care services plan: 2011–2015].

[11] Turner T, Misso M, Harris C, Green S (2008). Development of evidence-based clinical practice guidelines (CPGs): comparing approaches. Implement Sci.

[12] Metoder og verktøy [Methods and tools]. In: https://helsedirektoratet.no/metoder-og-verktoy. Accessed 21 Mar 2017.

[13] Grant BF, Stinson FS, Dawson DA, Chou SP, Dufour MC, Compton W, Pickering RP, Kaplan K (2004). Prevalence and co-occurrence of substance use disorders and independent mood and anxiety disorders: results from the National Epidemiologic Survey on alcohol and related conditions. Arch Gen Psychiatry.

[14] Morisano D, Babor T, Robaina K (2014). Co-occurrence of substance use disorders with other psychiatric disorders: implications for treatment services. Nordic Stud Alcohol Drugs.

[15] Landheim AS, Bakken K, Vaglum P (2003). Gender differences in the prevalence of symptom disorders and personality disorders among poly-substance abusers and pure alcoholics. Substance abusers treated in two counties in Norway. Eur Addict Res.

[16] Lai HM, Cleary M, Sitharthan T, Hunt GE (2015). Prevalence of comorbid substance use, anxiety and mood disorders in epidemiological surveys, 1990-2014: a systematic review and meta-analysis. Drug Alcohol Depend.

[17] Saban A, Flisher AJ (2010). The association between psychopathology and substance use in young people: a review of the literature. J Psychoactive Drugs.

[18] Helsedirektoratet [The Norwegian Directorate of Health]. Nasjonal faglig retningslinje for utredning, behandling og oppfølging av personer med samtidig rus- og psykisk lidelse - ROP-lidelser [national guideline for assessment, treatment and social rehabilitation of persons with concurrent substance use disorders and mental disorders]. Oslo: The Norwegian Directorate of Health; 2012.

[19] Gap-undersøkelse for behandlere i psyksik helsevern [Audit-survey for health professionals in specialist mental health services]. Nasjonal kompetansetjeneste for samtidig rusmisbruk og psykisk lidelse [Norwegian National Advisory Unit on concurrent substance abuse and mental health]. In: http://gap.rop.no/skjemamaler/for-behandlere-i-psykisk-helsevern.-versjon-2.0. Accessed 09 Sept 2015.

[20] Sheldon TA, Cullum N, Dawson D, Lankshear A, Lowson K, Watt I, West P, Wright D, Wright J. What's the evidence that NICE guidance has been implemented? Results from a national evaluation using time series analysis, audit of patients’ notes and interviews. BMJ. 2004;329(7473):999 10.1136/bmj.329.7473.999 PMC524545 15514342

[21] Francke AL, Smit MC, de Veer AJ, Mistiaen P (2008). Factors influencing the implementation of clinical guidelines for health care professionals: a systematic meta-review. BMC Med Inform Decis Mak..

[22] Boaz A, Baeza J, Fraser A (2011). Effective implementation of research into practice: an overview of systematic reviews of the health literature. BMC Res Notes.

[23] Grol R, Wensing M, Eccles M (2005). Improving patient care: the implementation of change in clinical practice.

[24] Grimshaw J, Eccles M, Thomas R, MacLennan G, Ramsay C, Fraser C, Vale L (2006). Toward evidence-based quality improvement - evidence (and its limitations) of the effectiveness of guideline dissemination and implementation strategies 1966-1998. J Gen Intern Med.

[25] Nilsen P (2015). Making sense of implementation theories, models and frameworks. Implement Sci.

[26] Barbui C, Girlanda F, Ay E, Cipriani A, Becker T, Koesters M (2014). Implementation of treatment guidelines for specialist mental health care. Cochrane Database Syst Rev.

[27] The Effective Practice and Organisation of Care (EPOC) Group. http://epoc.cochrane.org/. Accessed 20 Mar 2017.

[28] Foy R, Eccles M, Straus SE, Tetroe J, Graham ID (2009). Audit and feedback interventions. Knowledge translation in health care: moving from evidence to practice.

[29] Ivers NM, Sales A, Colquhoun H, Michie S, Foy R, Francis JJ, Grimshaw JM (2014). No more ‘business as usual’ with audit and feedback interventions: towards an agenda for a reinvigorated intervention. Implement Sci.

[30] Ivers NM, Grimshaw JM, Jamtvedt G, Flottorp S, O'Brien MA, French SD, Young J, Odgaard-Jensen J (2014). Growing literature, stagnant science? Systematic review, meta-regression and cumulative analysis of audit and feedback interventions in health care. J Gen Intern Med.

[31] Hysong SJ, Best RG, Pugh JA (2006). Audit and feedback and clinical practice guideline adherence: making feedback actionable. Implement Sci.

[32] van der Veer S, de Keizer N, Ravelli A, Tenkink S, Jager K (2010). Improving quality of care. A systematic review on how medical registries provide information feedback to health care providers. Int J Med Inform.

[33] Baxter P, Jack S (2008). Qualitative case study methodology: study design and implementation for novice researchers. Qual Rep.

[34] Stake RE (1995). The art of case study research.

[35] Graham ID, Straus SE, Tetroe J (2009). Knowledge translation in health care: moving from evidence to practice.

[36] Velkommen til GAP [Welcome to GAP]. In: http://gap.rop.no. Accessed 25 Jan 2018.

[37] Braun V, Clarke V (2006). Using thematic analysis in psychology. Qual Res Psychol.

[38] Stake RE (2006). Multiple case study analysis.

[39] Yin RK (2014). Case study research : design and methods.

[40] Kristensen H, Hounsgaard L (2014). Evaluating the impact of audits and feedback as methods for implementation of evidence in stroke rehabilitation. Br J Occup Ther.

[41] Sinuff T, Muscedere J, Rozmovits L, Dale CM, Scales DC (2015). A qualitative study of the variable effects of audit and feedback in the ICU. BMJ Qual Saf.

[42] Taylor A, Neuburger J, Walker K, Cromwell D, Groene O. How is feedback from national clinical audits used? Views from English National Health Service trust audit leads. J Health Serv Res Policy. 2016; 10.1177/1355819615612826. 10.1177/1355819615612826 26811374

[43] Davis DA, Mazmanian PE, Fordis M, Van Harrison R, Thorpe KE, Perrier L (2006). Accuracy of physician self-assessment compared with observed measures of competence - a systematic review. JAMA.

[44] Statens helstilsyn/Norwegian Board of Health Supervision. Tilsynsmelding 2016 [supervision report 2016]. In: https://www.helsetilsynet.no/no/Publikasjoner/Tilsynsmelding/Tilsynsmelding-2016/. Accessed 16 Mar 2017.

[45] Norsk pasientskadeerstatning/the Norwegian system of patient injury compensation. Årsrapport 2016 [annual report 2016]. In. https://www.npe.no/globalassets/dokumenter-pdf-og-presentasjoner/rapporter/npe_arsrapport_2016_web.pdf. Accessed 17 Mar 2017.

[46] Gagliardi AR, Brouwers MC, Finelli A, Campbell CM, Marlow BA, Silver IL (2011). Physician self-audit: a scoping review. J Contin Educ Heal Prof.

[47] Broyles LM, Rodriguez KL, Kraemer KL, Sevick MA, Price PA, Gordon AJ (2012). A qualitative study of anticipated barriers and facilitators to the implementation of nurse-delivered alcohol screening, brief intervention, and referral to treatment for hospitalized patients in a veterans affairs medical center. Addict Sci Clin Pract.

[48] Forsner T, Hansson J, Brommels M, Wistedt AA, Forsell Y (2010). Implementing clinical guidelines in psychiatry: a qualitative study of perceived facilitators and barriers. BMC Psychiatry.

[49] Cabana MD, Rand CS, Powe NR, Wu AW, Wilson MH, Abboud PA, Rubin HR (1999). Why don't physicians follow clinical practice guidelines? A framework for improvement. JAMA.

[50] MacNaughton K, Chreim S, Bourgeault IL (2013). Role construction and boundaries in interprofessional primary health care teams: a qualitative study. BMC Health Serv Res.

[51] Versteeg M, Laurant M, Franx G, Jacobs A, Wensing M (2012). Factors associated with the impact of quality improvement collaboratives in mental healthcare: an exploratory study. Implement Sci.

[52] Michie S, Pilling S, Garety P, Whitty P, Eccles MP, Johnston M, Simmons J (2007). Difficulties implementing a mental health guideline: an exploratory investigation using psychological theory. Implement Sci.

[53] Del Fiol G, Workman TE, Gorman PN (2014). Clinical questions raised by clinicians at the point of care: a systematic review. JAMA Intern Med.

[54] Clarke MA, Belden JL, Koopman RJ, Steege LM, Moore JL, Canfield SM, Kim MS (2013). Information needs and information-seeking behaviour analysis of primary care physicians and nurses: a literature review. Health Inf Libr J.

[55] O’leary DF, Mhaolrúnaigh SN (2012). Information-seeking behaviour of nurses: where is information sought and what processes are followed?. J Adv Nurs.

[56] Weng Y, Kuo KN, Cy Y, Lo H, Yh S, Yw C (2013). Information-searching behaviors of main and allied health professionals: a nationwide survey in Taiwan. J Eval Clin Pract.

[57] Horsfall J, Cleary M, Hunt GE, Walter G (2009). Psychosocial treatments for people with co-occurring severe mental illnesses and substance use disorders (dual diagnosis): a review of empirical evidence. Harv Rev Psychiatry.

[58] Haggerty JL, Reid RJ, Freeman GK, Starfield BH, Adair CE, McKendry R (2003). Continuity of care: a multidisciplinary review. BMJ.

[59] Reid RJ, Wagner EH (2008). Strengthening primary care with better transfer of information. CMAJ.

[60] Danbolt LJ, Kjönsberg K, Lien L (2010). Hjelp når du trenger det: en kvaliatativ studie av samhandling og gjensidifhetskunnskap i den psykiske helsetjenesten [help when you need it. A qualitative study of the interactions and mutual knowledge between primary and secondary level functions in psychiatric health care]. Tidsskrift for Psykisk Helsearbeid.

[61] Fredheim T, Danbolt LJ, Haavet OR, Kjonsberg K, Lien L (2011). Collaboration between general practitioners and mental health care professionals: a qualitative study. Int J Ment Health Syst.

[62] Fickel JJ, Parker LE, Yano EM, Kirchner JE (2007). Primary care - mental health collaboration: an example of assessing usual practice and potential barriers. J Interprof Care.

[63] Shiffman RN, Dixon J, Brandt C, Essaihi A, Hsiao A, Michel G, O'Connell R (2005). The GuideLine Implementability appraisal (GLIA): development of an instrument to identify obstacles to guideline implementation. BMC Med Inform Decis Mak.

[64] Creswell JW (2013). Qualitative inquiry & research design : choosing among five approaches.

