# Supplementary material for: Acting on audit & feedback: a qualitative instrumental case study in mental health services in Norway
Source: BMC Health Serv Res. 2018 Jan 31;18:71. doi: 10.1186/s12913-018-2862-y (PMC5793343; doi:10.1186/s12913-018-2862-y)
Supplement: Additional file 1: — Information on The National Guideline for Assessment, Treatment and Social Rehabilitation of Persons with Concurrent Substance Use and Mental Health Disorders. (DOCX 16 kb) [file 12913_2018_2862_MOESM1_ESM.docx]

**Information on The National Guideline for Assessment, Treatment and Social Rehabilitation of Persons with Concurrent Substance Use and Mental Health Disorders**

“The National Guideline for Assessment, Treatment and Social Rehabilitation of Persons with Concurrent Substance Use and Mental Health Disorders” was launched in March 2012. A working group consisting of health professionals from primary and specialist services in mental health and addiction and representatives from patient organisations developed the guideline. It consists of 93 recommendations in the areas of user participation, assessment, treatment and follow-up, and roles and responsibilities, with a separate chapter on implementation. The evidence of the recommendations was graded by using the SIGN classification (Scottish Intercollegiate Guidelines Network). The National Guideline was developed for healthcare providers in severe and less severe mental disorders and substance use in primary and specialist services. It was targeted at people with severe and less severe mental illness combined with substance use disorder when each of the two types of disorder is associated with significant impairment. The National Guideline was assessed with AGREE-II and achieved a good quality score. Various initiatives for implementation were prepared as the guideline was launched, for example an audit survey, a web-site (www.snakkomrus.no), and a national learning program. It has been published in paper and as an electronic version linked to for example the recommended screening tools. A user panel chose the ten most important recommendations to be published as a pamphlet, poster, and in paper and electronic versions to support implementation.

Areas and example of recommendations in The National Guideline for Assessment, Treatment and Social Rehabilitation of Persons with Concurrent Substance Use and Mental Health Disorders

| Area | Example of recommendations |
| --- | --- |
| User involvement | #5 Family members should as far as possible be involved in the treatment and follow-up care when the services user so wishes |
| Screening | #14 For screening for problematic alcohol use in people with a known mental disorder, it is recommended to use the tools CAGE or AUDIT  #15 For screening for problematic use of drugs or medicines in people with a known mental disorder, it is recommended to use the tool DUDIT |
| Diagnosis | #28 When it is suspected that a person has a concurrent substance use disorders and mental illness, a diagnostic evaluation of both substance use disorder and mental illness and the relationship between them must be done |
| Treatment | #49 Treatment that combines cognitive behavior therapy with motivational interviewing should be offered to those who need it |
| Roles and Responsibility | #84 Patients in specialist mental health services with less severe substance use disorders should be offered treatment for the disorder at the same place, possibly through cooperation with TSB (interdisciplinary specialized substance abuse treatment)  #85 Specialist mental health services should be able to provide treatment to patients with the combination of severe mental disorder and all types of substance use disorders |
